# Atomistic modeling of tribological properties of Pd and Al nanoparticles on a graphene surface

**DOI:** 10.3762/bjnano.9.115

**Published:** 2018-04-19

**Authors:** Alexei Khomenko, Miroslav Zakharov, Denis Boyko, Bo N J Persson

**Affiliations:** 1 Sumy State University, 40007 Sumy, Ukraine; 2 Peter Grünberg Institut-1, Forschungszentrum-Jülich, D-52425 Jülich, Germany

**Keywords:** aluminum, friction force, graphene, nanoparticle, nanotribology, palladium

## Abstract

**Background:** The frictional properties of nanoparticles have been studied to gain insight into the fundamental origin of sliding friction.

**Results:** Using molecular dynamics we investigate frictional properties of aluminum and palladium nanoparticles deposited on a graphene layer. We study the time evolution of the total momentum of the system, the total and potential energies, the temperature, the velocity and position of the center of mass, the dimensions of the nanoparticle, and the friction and substrate forces acting on the particle. We also study how the friction force depends on the nanoparticle–graphene contact area and the temperature.

**Conclusion:** The tribological properties of nanoparticles strongly depend on the materials. The particles move in an irregular (saw-like) manner. The averaged friction force depends nearly linearly on the contact area and non-monotonously on temperature. We observe ordered crystalline domains of atoms at the bottom surface of the metal particles, but the peaks of radial distribution function are blurred indicating that the nanoparticles are amorphous or polycrystalline.

## Introduction

The study of surface or interface phenomena at the atomic level has attracted considerable interest over the past four decades. This is due to the development of new experimental techniques, for example, atomic force microscope, dynamic friction force microscope, and owing to the continuous miniaturization of electronic and mechanical devices [1–14].

There are many studies concerning the tribological properties of nanoobjects. For example, alumina nanoparticles were studied in [9] and self-organized monolayers in [4]. In [5] the authors studied the interaction in ultrahigh vacuum between a nanoasperity and an alkali-metal halide surface at different temperatures, and showed how the static friction and contact stiffness depend on the contact area. They observed “contact aging” due to stress-aided, thermally activated atomic rearrangement processes.

The term “contact aging” [6] is related to time-dependent atomic reconstructions at the interface between the slider and the substrate, which usually lead to an increase in friction with time. Modern rate-and-state models for rough contacts predict that aging does not only influence the transition from static contact to sliding, but affects the overall sliding dynamics.

The complex nature of the friction dynamics of metallic nanoparticles [14] makes it virtually impossible to construct a general and reliable analytical theory of the phenomena under consideration. Therefore, computer modeling, in particular molecular dynamics (MD), is a useful tool for the theoretical study of friction and wear at the atomic level [2,8,10,12,15–19]. Preliminarily MD studies were carried out for the formation and friction of Ag, Ni, Au, Cu nanoparticles on graphene [10–11]. This paper extends the study to Al and Pd nanoparticles [17]. Besides, in previous papers the temperature dependence of the friction force is not investigated. In the manuscript here, the contact area of nanoparticles on graphene is calculated more precisely using a new method.

Classical MD algorithms can be found in [12]. Although MD simulations of friction at the atomic level have provided some understanding of interfacial processes, they are limited to much shorter spatial and temporal length scales than in most experiments. Recent experimental and computer simulation studies of static and sliding friction of metallic nanoparticles have focused on the dependence of friction on the particle size, morphology and orientation [10–11,13–14,17].

For atomistic modeling the material-dependent potential energy is needed. Here we use empirical potentials where the potential energy can be represented as a function of the atomic positions. For aluminum and palladium an empirical many-body potential is employed based on the embedded atom method [15]. It is designed to model alloys and is fully expressed through analytical functions, unlike the first versions of the embedded atom method, where cubic splines were used for the embedding function. The in-plane forces in graphene are described by a spring potential, and the interactions between the nanoparticle and the graphene carbon atoms are taken as a Lennard-Jones potential, which was chosen the same for the Al and Pd atoms [10–11].

The MD method differs from most experimental studies in that usually the total energy *E* and volume *V* are fixed, while the temperature *T* and the pressure *P* fluctuate. In terms of statistical mechanics, conventional MD yields quantities averaged over the microcanonical ensemble *NVE* (*N* is the number of molecules) [12], but experiments with a constant temperature correspond to the canonical ensemble *NVT*. Friction and wear phenomena are usually accompanied by local heating of the interface, which occurs as the result of work done on the system (in our problem this work is done by the external force, which is assumed to move the nanoparticle relatively to the graphene sheet). To dissipate this excess heat in the MD simulations any one of a large number of available thermostats is used. Velocity rescaling by a constant factor, which corresponds to the desired temperature, is the simplest way to maintain the necessary temperature. Here we use the Berendsen thermostat [16–17] that does not give the trajectory of the true canonical ensemble and account for numerical and round errors.

## Results and Discussion

**Figure 1:**
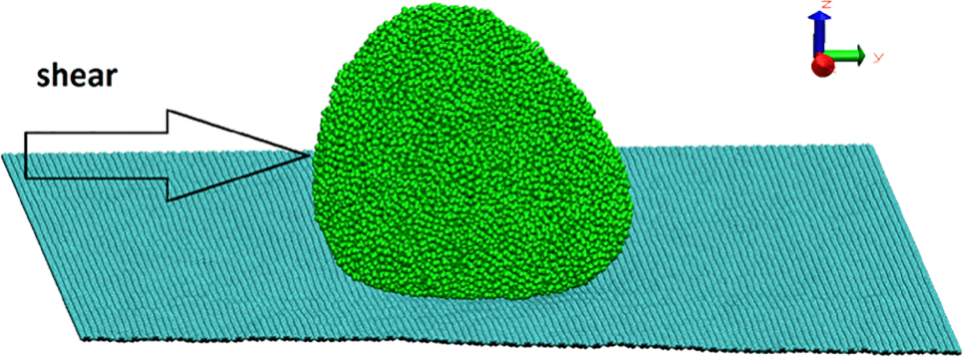
Perspective snapshot of the formed nanoparticle containing 20000 Al atoms.

**Figure 2:**
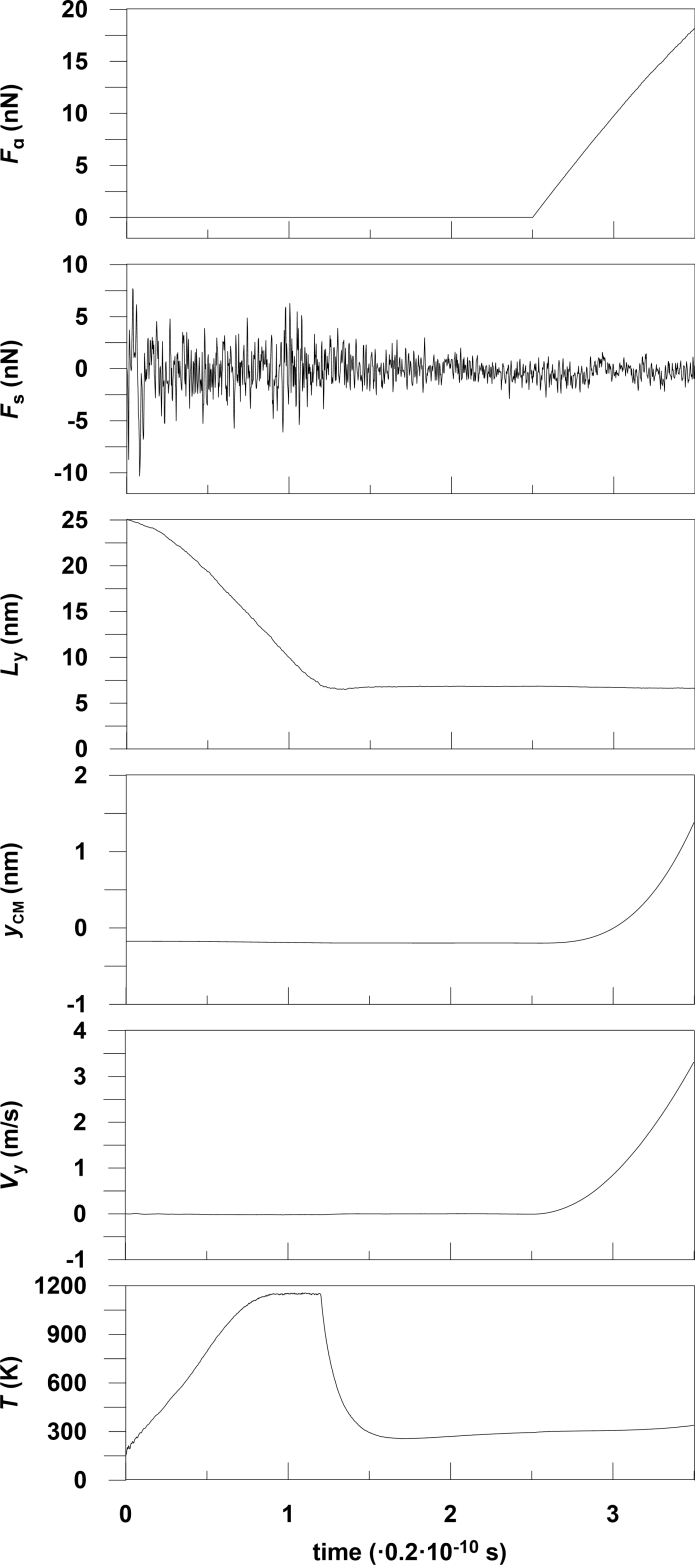
Time dependencies of temperature *T* of the system, velocity *V_y_* and lateral position *y_CM_* of the center of mass of the nanoparticle, lateral size *L_y_*, total substrate force *F_s_*, applied force *F_a_* calculated for Pd layer and nanoparticle containing 20000 atoms.

We have performed MD calculations for the sliding of Al and Pd metallic nanoparticles on graphene. The lateral sizes *L_x_*, *L_y_*, *L_z_* of metal nanoparticles along the *x*,*y*,*z*-axes have been calculated as the difference between the coordinates of metal atoms with maximum and minimum values along the *x*,*y*,*z*-axes. The substrate in our model is a graphene sheet that is parallel to the *xy*-plane with armchair and zigzag fixed edges along the *x*- and *y*-directions (Figure 1). Snapshots in this paper were taken by using the Visual Molecular Dynamics software. The maximum velocity of the Al and Pd nanoparticles consisting of 20000 atoms equals 9.83 m/s and 3.31 m/s, respectively. The metal atoms are first placed as a thin slab with face-centered cubic lattice structure above the graphene, but quickly rearrange into more compact conformation corresponding to a state with lower free energy, but most likely not the free-energy minimum state. This is manifested in Figure 2 in the decrease of *L_y_* with increasing time, and as a strong increase in the temperature *T*, but the Berendsen thermostat finally results in the imposed temperature.

**Figure 3:**
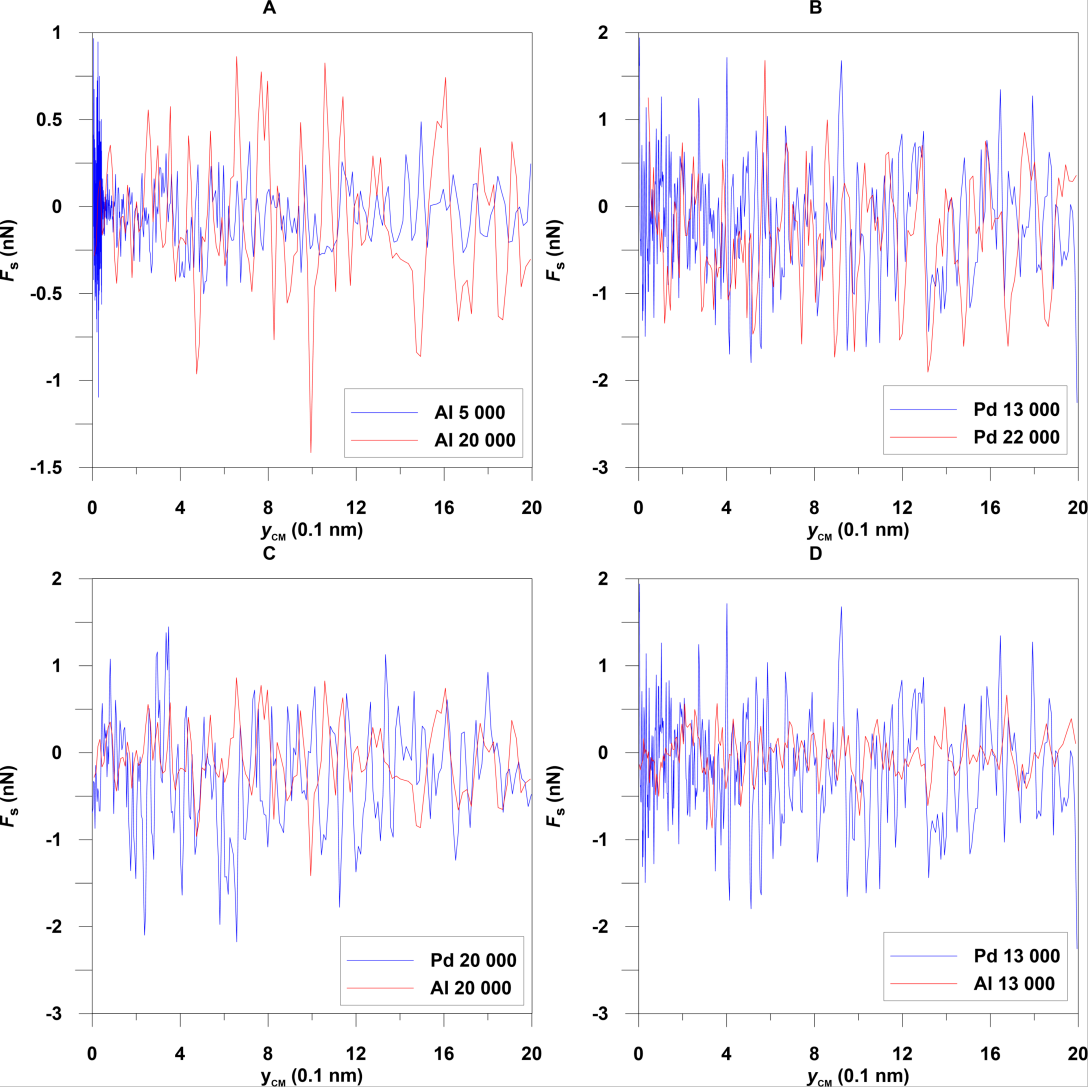
Substrate force as a function of the lateral position of the center of mass of the nanoparticles: (A) Al with 5000 and 20000 atoms, (B) Pd with 13000 and 22000 atoms, (C) Al and Pd with 20000 atoms, (D) Al and Pd with 13000 atoms at a temperature of 300 K.

Next, the Berendsen thermostat is turned off, and an external driving force *F_a_* is applied to the nanoparticle. In manipulation experiments with atomic force microscopes pushing is always involved. Pushing in our system is simulated by applying a force *F_a_* along the *y*-direction uniformly distributed on all metal atoms with values of *y*-coordinates less than the *y*-coordinate of the center of mass of the nanoparticle. Therefore the nanoparticle moves (on the average) along the *y*-axis. The maximum applied force for the Al and Pd particles is 40 nN and 18.14 nN, respectively. The substrate force *F_s_* is the sum of the *y*-components of forces acting on Al and Pd atoms from the graphene atoms. The force *F_s_* varies irregularly with time and has a sawtooth form, which is associated with stick-slip motion of the nanoparticle Figure 3. Figures for the characteristics of Al nanoparticles are represented in [17].

**Figure 4:**
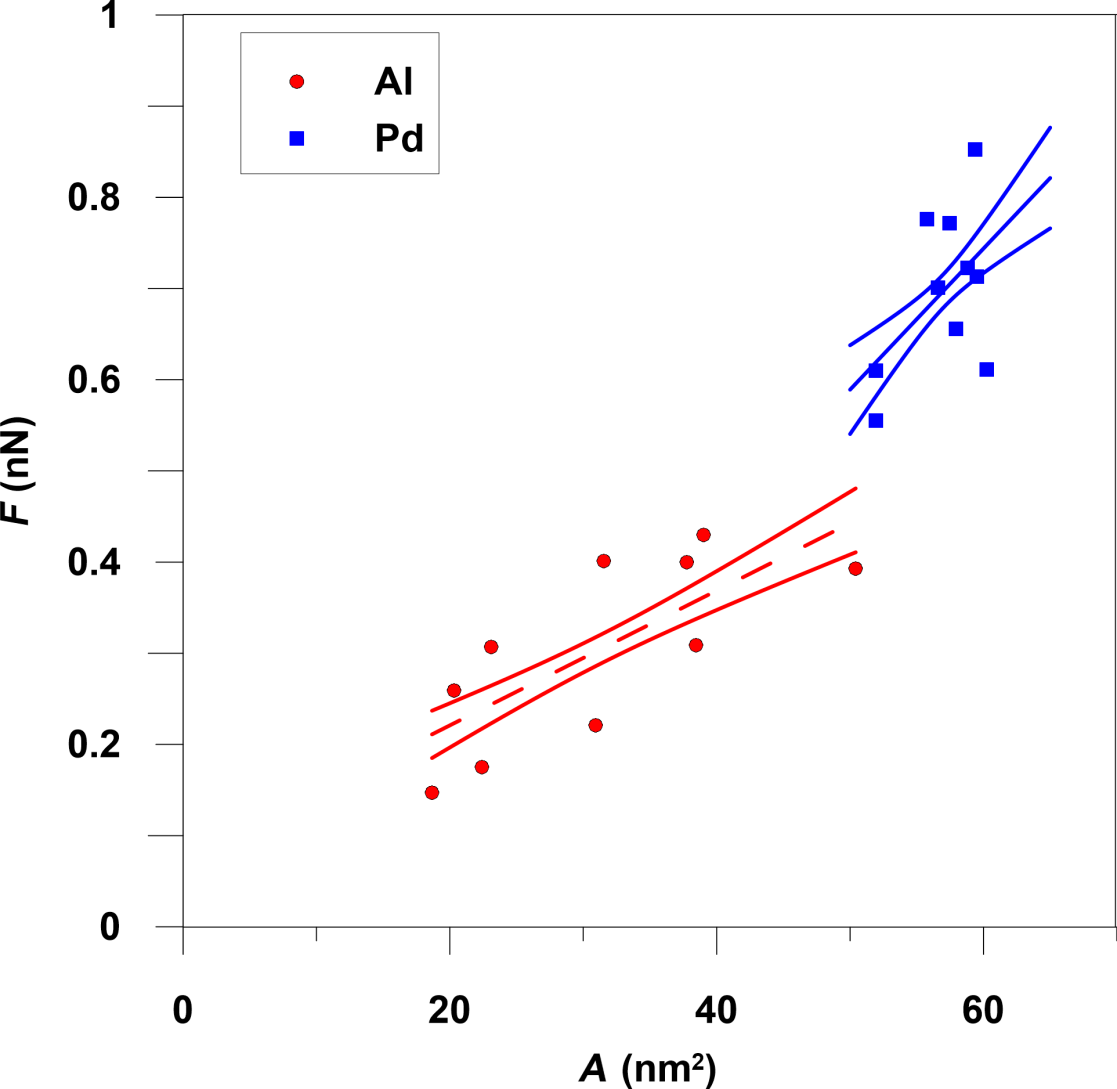
Friction force as a function of the contact area with confidence intervals of the approximation at a temperature of 300 K. The linear approximations for the slopes of the average friction force for aluminum and palladium are 0.007 nN/nm^2^ and 0.015 nN/nm^2^, respectively.

**Figure 5:**
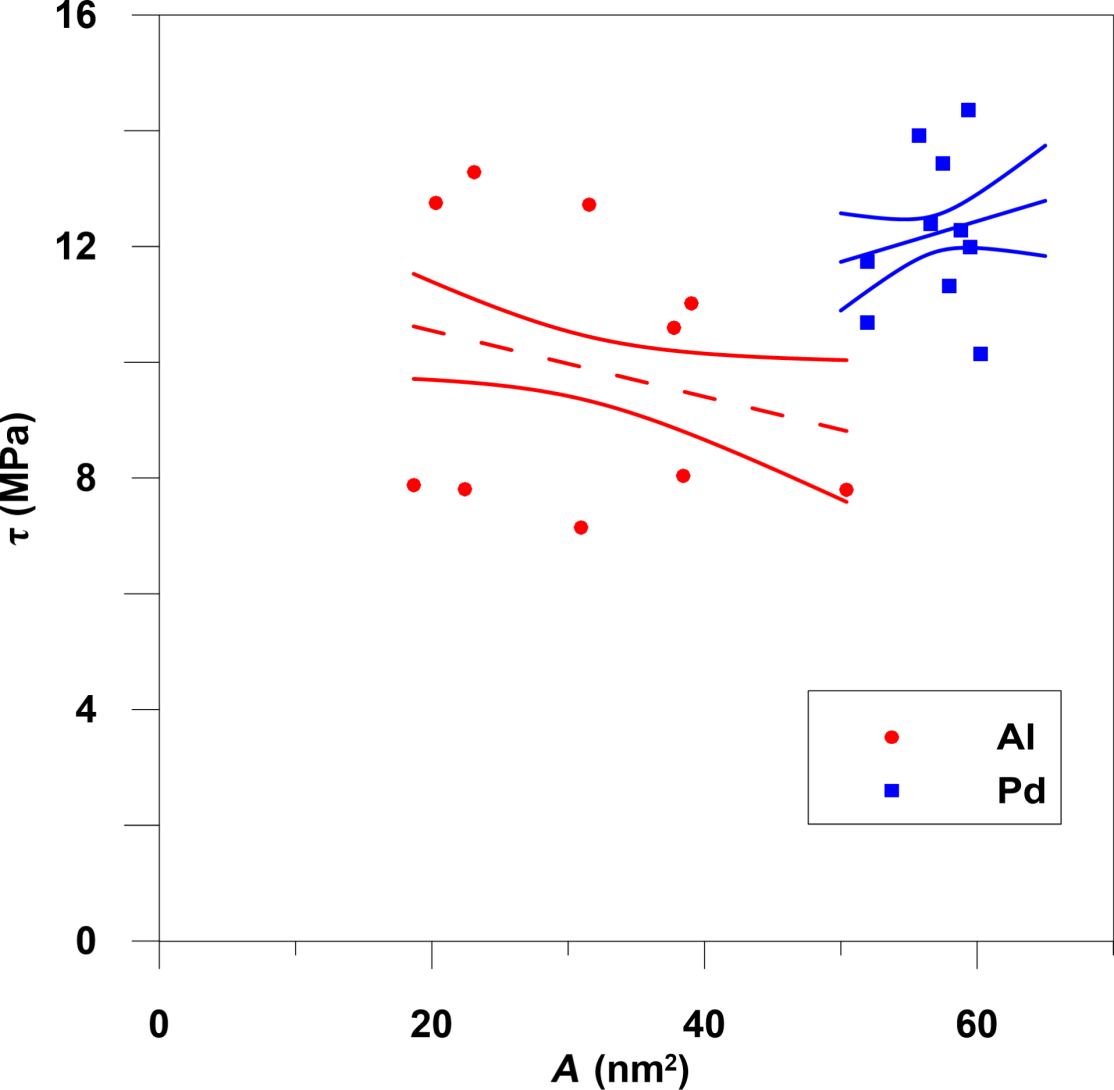
Shear stress as a function of the contact area with confidence intervals of the approximation at a temperature 300 K.

The dependence of the friction force on the contact area has been calculated for Al nanoparticles (formed from 5000–20000 atoms) and for palladium nanoparticles (formed from 13000–22000 atoms). Figure 4 shows that the friction force increases approximately linearly with contact area. The friction forces are averaged over the total simulation time period excluding the formation step. Each point in Figure 4 is the result of averaging 20–30 measurements of substrate force and contact area at different time steps during movement. Different points of the same color correspond to different number of Al or Pd atoms. Figure 5 depicts the frictional shear stress τ as a function of the contact area. The average shear stress is τ ≈ 9.9 MPa for the Al particles and 12.2 MPa for Pd particles. The values of shear stress in experiments (Figure 2 in [7]) for Sb particles on MoS_2_ substrate varies from 1 MPa to 3 MPa with contact area changes from 1000 nm^2^ to 100000 nm^2^. Also, for Sb particles on highly oriented pyrolytic graphite τ is in the range from 0.1 MPa to 1 MPa with the same changes of contact area. In [7] the shear stress decreases linearly with contact area while we find that τ is nearly independent of it. This may result from the different sizes of the contact area, namely 10^3^–10^5^ nm^2^ in experiments versus ca. 10 nm^2^ in our simulations, and different interaction potentials, and different length and time scales.

**Figure 6:**
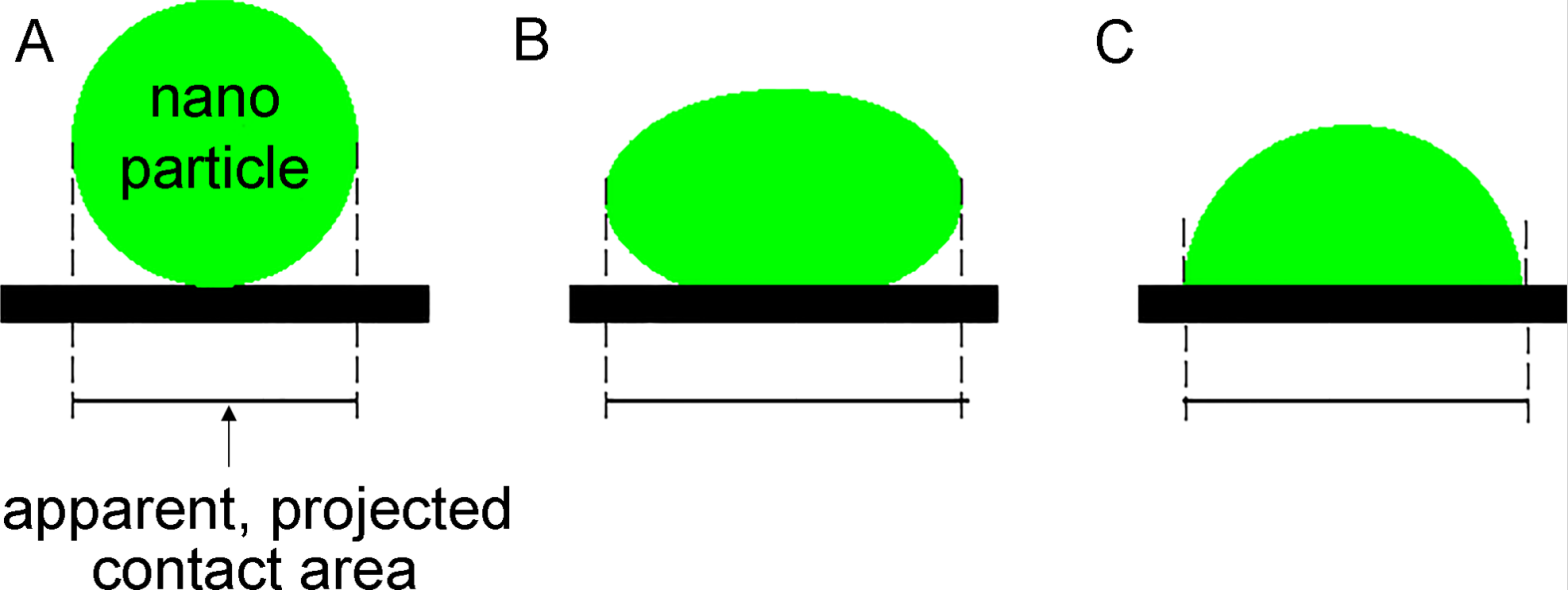
A nanoparticle (green) on a substrate (black) when the interaction between the particle and the substrate is (A) very weak, (B) of intermediate strength and (C) strong.

**Figure 7:**
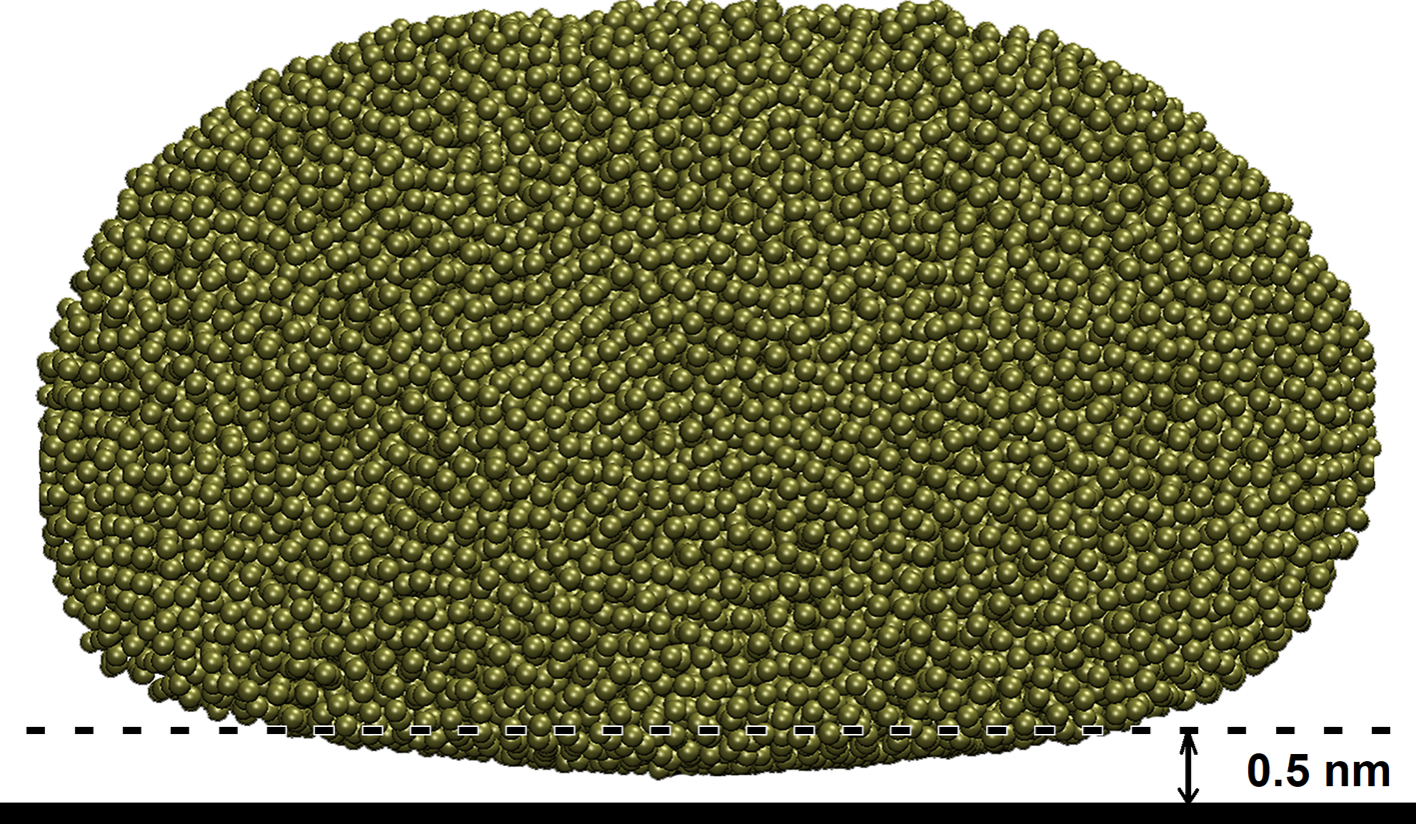
Side view of a Pd nanoparticle with 20000 atoms (the horizontal solid line is the graphene plane) at a temperature of 300 K.

The contact area in [10–11] is determined, as in experiments, on the basis of the lateral sizes *L_x_* and *L_y_* of the nanoparticle, approximating it with an ellipse. However, for particles where the internal bonding potential between the atoms is much stronger than the bonding potential to the substrate (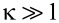), this approach may result in a “contact area” that is much bigger than the area obtained by only including those atoms that interact strongly with the substrate. This is illustrated in Figure 6. In Figure 6c the interaction potential to the substrate is large (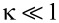), and the particle takes the shape of a spherical (or elliptic) cup. In this case the projected geometrical area is close to the area where the surface atoms interact strongly with the substrate. In the opposite limit (Figure 6a), where the interaction potential with the substrate atoms is very weak (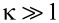), the particle takes a nearly spherical shape and in this case the projected area, as would be obtained using, e.g., atomic force microscopy, would be much larger than the surface area where strong interaction with the substrate occurs. In our study we are in an intermediate interaction potential region where the contact appears as in Figure 6b. In this case using the projected contact area will not give the area where the interaction between the atoms of the particle and the substrate is strong. In our previous investigations [10–11], the contact area was calculated by using an ellipse. This approach is quite inaccurate because most nanoparticles do not have ideal forms of ellipsoids or spheres. In this study the contact area is defined as the sum of atomic contact areas of all metal atoms of which the distance from their centers to the centers of carbon atoms is below than 0.5 nm (Figure 7).

**Figure 8:**
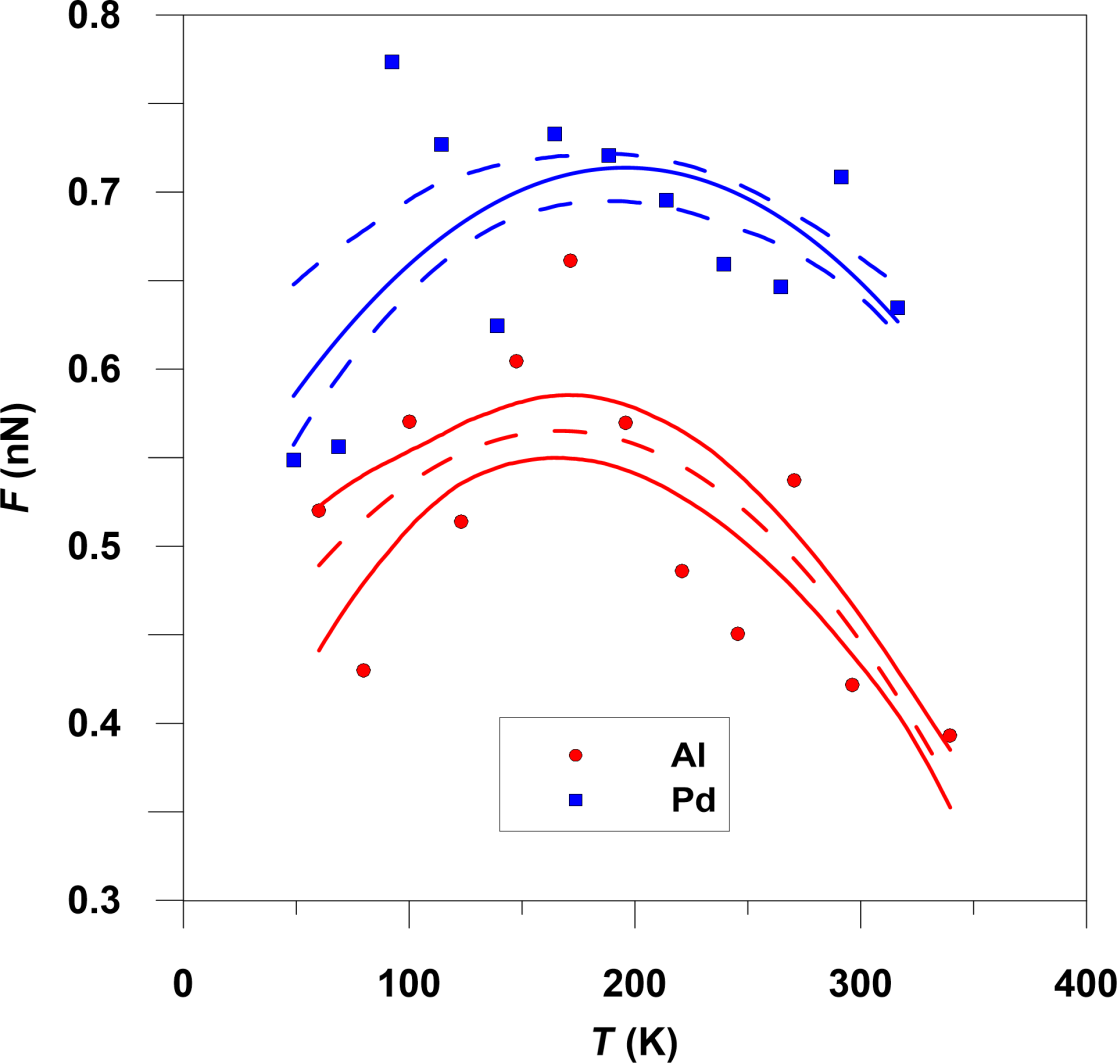
Friction force versus temperature with confidence intervals of the second-degree polynomial approximation.

Figure 8 shows the temperature dependence of the friction force. The contact area almost does not change with the temperature. The maximal temperature, which we explore, corresponds to the room temperature at standard atmosphere pressure and processes such as melting do not occur. The friction force in both cases first increases, and then reaches a maximum at *T* ≈ 170 K. Note that the Pd particles exhibit larger friction than the Al particles, even when the contact area with the graphene is the same. This results from the different lattice constant and atomic arrangements in Al and Pd particles. A similar non-monotonous behavior of friction is observed for hexadecanethiol self-assembled monolayers on Au substrates [4] and NaCl(001) crystal surfaces in ultrahigh vacuum [5].

The temperature dependence of the friction shown in Figure 8 can be understood as follows [20–23]: At high temperatures the friction decreases with increasing temperature due to thermal fluctuations, which help to move the particles over the energy barriers they experience during sliding. These are usually denoted as thermally activated, stress-aided, processes. At low temperature the friction force may decrease with decreasing temperature. This is due to the fact that thermal excitations (thermally excited fluctuations) are necessary in order for the particle to “find” configurations of large binding energy along the sliding path. As a result, at low temperature the interface will become more incommensurate, resulting in an interaction potential that exhibits a smaller energy corrugation along the sliding path and lower friction. Thus, we expect the friction force to exhibit a maximum at a characteristic temperature (that increases with increasing sliding speed).

**Figure 9:**
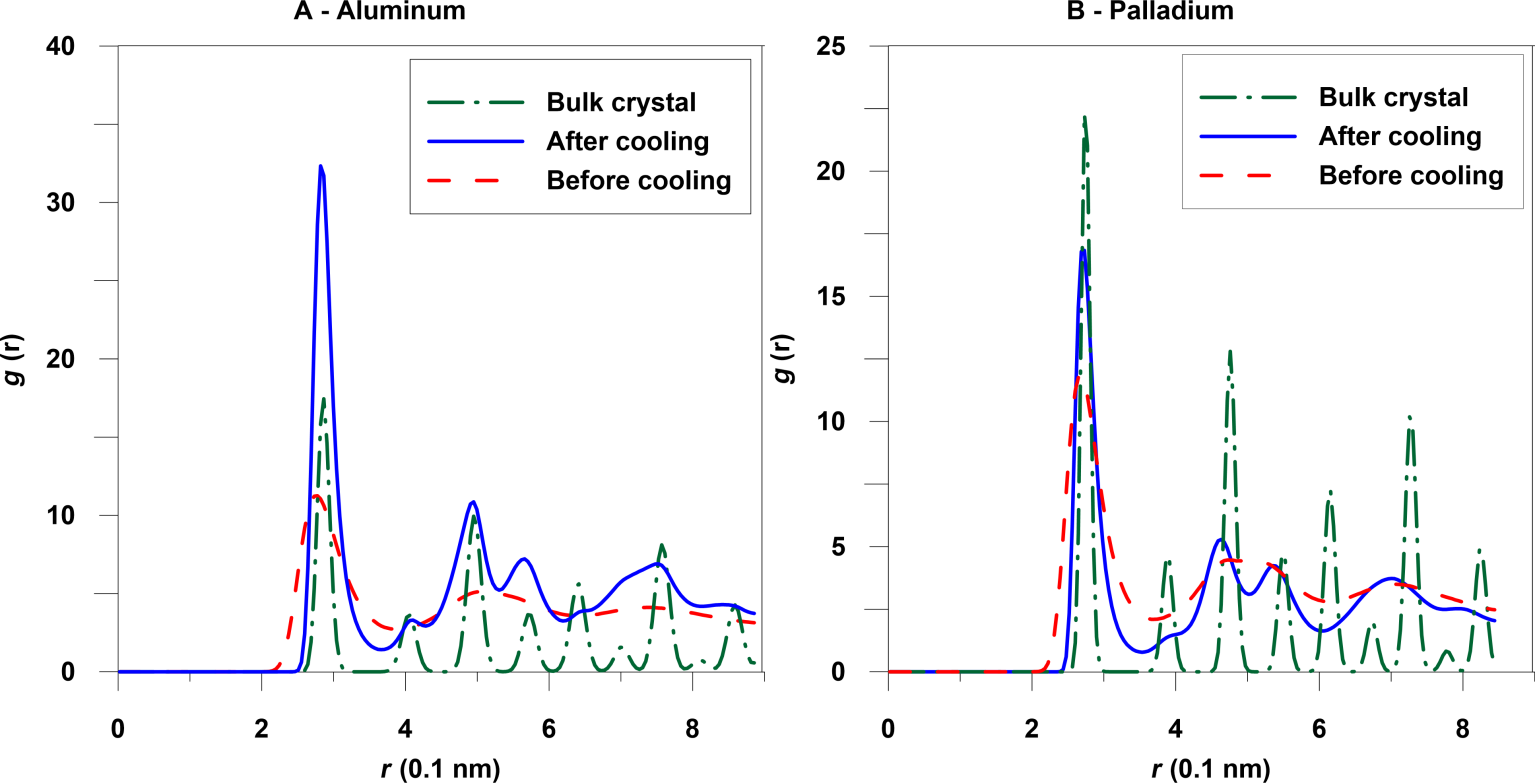
Radial distribution function for the Al and Pd nanoparticles containing 20000 atoms for the bulk crystal at a temperature of 300 K and after cooling, and at 1150 K for palladium before cooling, and at 750 K for aluminum before cooling.

The structure of the nanoparticles has been analyzed by using the radial distribution function (RDF) (Figure 9). When the nanoparticle has been formed, the RDF displays blurred peaks reflecting its disordered structure. After cooling, some ordering occurs resulting in a higher first peak in the RDF, which is located at the nearest-neighbor distance in the bulk state (2.863 Å for Al and 2.729 Å for Pd). Nonetheless, the peaks are much broader than for the ideal bulk crystal, indicating amorphous or polycrystalline structures of nanoparticles.

**Figure 10:**
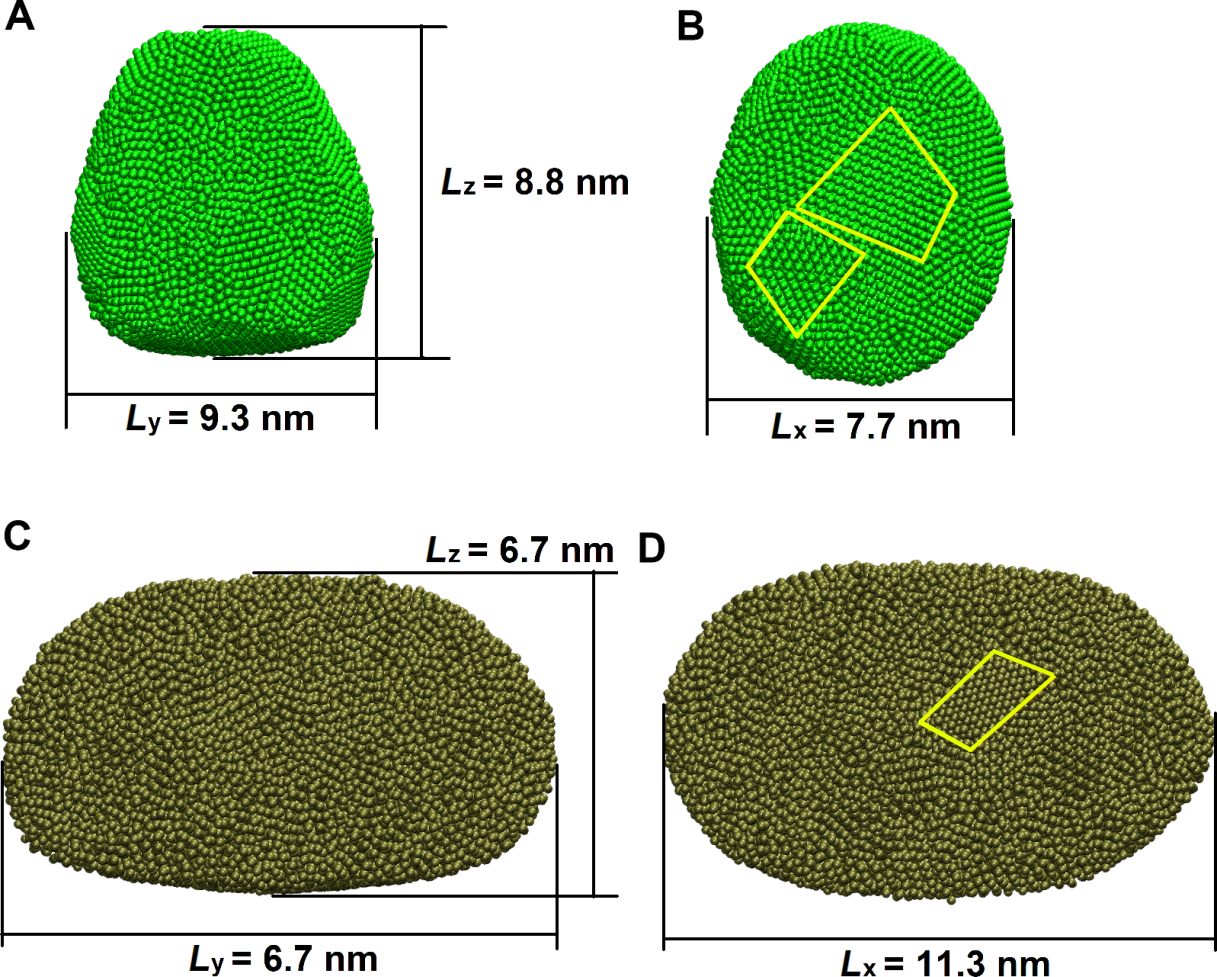
(A, C) Side and (B, D) bottom views of (A, B) Al and (C, D) Pd nanoparticles containing 20000 atoms at a temperature of 300 K. Nanoislands with local order are outlined by yellow contours.

Figure 10B,D shows the atoms of the Al and Pd nanoparticles at the particle–graphene interface. Note that domains of ordered atoms structures can be observed for both types of particles, but are more clear for the Al particle. Since the internal atom–atom bonding in the metallic clusters is much stronger (in particular for the Pd cluster) than the interaction potential between a metal atom and the graphene carbon atoms, it is likely that the ordered domains of metal atoms are not commensurate with the graphene lattice at any point during slip. Nevertheless, different orientations and positions of the particles on the graphene surface will generate different interaction energies with the graphene surface, and are the origin of the irregular stick-slip like motion of the nanoparticles [6,10–11,13,18–19,24–25].

## Conclusion

We have shown that the tribological properties of nanoparticles depend on the material. It was noted that the substrate force that acts on the nanoparticle has a sawtooth form as a function of coordinate (and time) as is also observed in nanotribological experiments [6,13–14]. We have studied the temperature dependence of friction, which is similar to that of hexadecanethiol self-assembled monolayers on Au substrates [4] and NaCl crystal surfaces in ultrahigh vacuum [5].

We found that the friction force, i.e., the force acting on the particles from the substrate, depends nearly linearly on the contact area consistent with [13–14]. The peaks of RDF are blurred and we can conclude that long-range atomic order is absent and the nanoparticles are amorphous or have a polycrystalline order. We have observed regions of local order of atoms on the particle bottom surface, which may influence the saw-like form of the substrate force as a function of time [6,10–11,13,18–19,24–26].

Previously we studied friction of Ag, Ni, Au and Cu nanoparticles on graphene [10–11]. In the present paper, we chose Al and Pd nanoparticles because the software we use allows to explore only metals with a face-centered cubic lattice. Besides, during testing the different metals, it was found that not all metallic atoms are assembled into spherical or ellipsoid nanoislands. In addition, for the selected amounts of 5000–25000 atoms not all metals have commensurate contact areas.

At a temperature of 300 K friction force depending on the contact area of Ni nanoparticles changes from 0.2 nN to 0.45 nN and from 0.1 nN to 0.2 nN for Ag, with contact area *A* from 20 nm^2^ to 60 nm^2^ for Ni and from 30 nm^2^ to 80 nm^2^ for Ag. The shear stress depending on the contact area of Ag nanoparticles varies from 40 MPa to 90 MPa and from 50 MPa to 140 MPa for Ni. The friction force depending on *A* of Au nanoparticles changes from 0.05 nN to 0.3 nN and from 0.1 nN to 0.25 nN for Cu, with *A* changing from 25 nm^2^ to 80 nm^2^ for Au and from 20 nm^2^ to 50 nm^2^ for Cu. The shear stress depending on the contact area of Au nanoparticles varies from 55 MPa to 95 MPa and from 60 MPa to 120 MPa for Cu. The contact area for shear stress measurement is the same as for friction force measurements. Thus, for all six kinds of investigated nanoparticles friction force and shear stress as a function of contact area change within these orders of magnitude. Only for Cu the linear approximation of friction force as a function of the contact area decreases and the linear approximation of shear stress as a function of *A* only decreases for Al. All other analogous approximations are increasing.
